# Early pre- and postsynaptic decrease in glutamatergic and cholinergic signaling after spinalization is not modified when stimulating proprioceptive input to the ankle extensor α-motoneurons: Anatomical and neurochemical study

**DOI:** 10.1371/journal.pone.0222849

**Published:** 2019-09-26

**Authors:** Kamil Grycz, Anna Głowacka, Benjun Ji, Julita Czarkowska-Bauch, Olga Gajewska-Woźniak, Małgorzata Skup

**Affiliations:** Nencki Institute of Experimental Biology, Warsaw, Poland; UCSF Weill Institute for Neurosciences, UNITED STATES

## Abstract

Alpha-motoneurons (MNs) innervating ankle extensor muscles show reduced peripheral inputs from Ia proprioceptive afferents and cholinergic afferents after chronic spinalization (SCT). That phenomenon is not observed on ankle flexor MNs, indicating a smaller vulnerability of the latter MNs circuit to SCT. Locomotor training of spinal rats which partially restored those inputs to extensor MNs tended to hyper innervate flexor MNs, disclosing a need for selective approaches. In rats with intact spinal cord 7-days of low-threshold proprioceptive stimulation of the tibial nerve enriched glutamatergic Ia and cholinergic innervation of lateral gastrocnemius (LG) MNs, suggesting usefulness of selective stimulation for restoration of inputs to extensor MNs after SCT. Accordingly, to examine its effectiveness after SCT, tibial nerves and soleus muscles were implanted bilaterally, and for MN identification fluorescence tracers to LG and tibialis anterior (TA) muscles were injected two weeks prior to spinalization. Stimulation of tibial nerve, controlled by H-reflex recorded in the soleus muscle, started on the third post-SCT day and continued for 7 days. Nine days post-SCT the number and volume of glutamatergic Ia and of cholinergic C-boutons on LG MNs was decreased, but stimulation affected neither of them. Postsynaptically, a threefold decrease of NMDAR *NR1* subunit and thirtyfold decrease of *M2* muscarinic receptor transcripts caused by SCT were not counteracted by stimulation, whereas a threefold decrease of AMPAR *GluR2* subunit tended to deepen after stimulation. We conclude that LG MNs, supported with proprioceptive stimuli after SCT, do not transcribe the perceived cues into compensatory response at the transcriptional level in the early post-SCT period.

## Introduction

Spontaneous reorganization of spinal circuit observed after complete spinal cord injury (SCT) includes changes of cholinergic innervation of the ankle extensor but not flexor MNs operating at the ankle joint muscles [1]. The number of cholinergic C-boutons abutting on the soma and proximal dendrites of soleus (Sol) MNs at six weeks after SCT was reduced by over 50% comparing to controls whereas those on tibialis anterior (TA) MNs were not changed [1]. Five weeks of treadmill locomotor exercise of spinal animals, begun one week after spinalization, partly reduced deficit in cholinergic innervation of Sol MNs but also tended to increase the number of C-boutons on TA MNs suggesting that this mode of activation of the spinal network will not restore the equilibrium between excitatory cholinergic inputs to these antagonistic groups of MNs [1]. Important conclusion, which emerged from that study, is that therapeutic methods aiming to bring functional improvement after SCT should be targeted to groups of MNs selectively impoverished after the damage. In our previous study we verified usefulness of selective treatment, by applying stimulation of low-threshold muscle afferents (predominantly Ia fibers) in the tibial nerve, controlled by means of Hoffmann (H) reflex, recorded in Sol muscle of the rats with the intact spinal cord. We hypothesized that it would bring enrichment of excitatory proprioceptive signaling to the ankle extensor MNs. Our assumption was that stimulation could reinforce not only Ia glutamatergic input to the extensor MNs innervated by this nerve branch but also exert widespread effect through spinal interneurons, including those terminating on the cholinergic V0_c_ group of neurons, the source of C-boutons abutting on MNs. Indeed, seven days of continuous bursts of low-threshold stimulation of proprioceptive fibers in the tibial nerve was effective not only in enrichment of glutamatergic Ia but also cholinergic terminals innervating the ankle extensor MNs in rats with intact spinal cord. Immunofluorescence (IF) labeling of vesicular transporter of glutamate, type 1 (VGLUT1), specific for monosynaptic Ia input to MNs [2–7], and of vesicular transporter of acetylcholine (VAChT), detecting C-boutons abutting on these MNs [1, 8], enabled evaluation of the effectiveness of that stimulation on terminal growth and on increased probability of neurotransmitter release [9]. Moreover, that stimulation caused a clear increase in expression of neurotrophin 3 (NT3) in the intact spinal cord and soleus muscle [10]. As the maintenance of glutamatergic Ia terminals on MNs [11, 12] and enhancement of their excitatory transmission depend on NT3 [13–15], the latter observation is a good predictor of synaptic modifications and compensatory effect on the low-threshold proprioceptive innervation of MNs under study.

To verify the effectiveness of stimulation of Ia afferents after SCT, in the current study we applied the same paradigm of stimulation starting on the third day after spinalization [9, 10]. Analysis of confocal images of synaptic terminals, identified by IF with antibodies against VGLUT1 and VAChT revealed that nine days after SCT a decline of Ia glutamatergic innervation and less profound decline of cholinergic innervation of LG MNs occured, but increased proprioceptive signaling did not affect neither of the terminal types. Because our electrophysiological data indicated that the responsiveness to Ia afferent stimulation in the H-reflex loop increased in time after SCT, and gene expression of numerous receptors in MNs was shown to undergo substantial transcriptional regulation in response to spinal cord injury [16–19], we asked whether spinalization impaired expression of glutamatergic and cholinergic receptors in this postlesion period and whether selective stimulation exerted postsynaptic changes in receptor expression in LG MNs. The response of TA MNs which did not undergo stimulation was investigated in parallel. The NMDA *NR1* subunit was chosen in the present study because it is mandatory to all NMDA receptors [20, 21], and AMPA *GluR2* subunit was chosen because AMPA receptors lacking that subunit become permeable to Ca(2+); the entrance of excess of this cation might be responsible for the vulnerability and maladaptive plasticity of spinal MNs [22]. Based on Mantilla’s Group reports [19, 23], we made assumption that if the stimulation exerted an increased MN responsiveness owing to receptor activity, mRNA expression of AMPA *GluR2* and NMDA *NR1* subunits would positively correlate with those responses after spinalization. A decrease of transcripts of NMDAR *NR1*, *AMPA GluR2* subunits and of *M2* muscarinic receptor, detected in microdissected LG and TA MNs after SCT was not compensated by stimulation.

We show here that one week of electrical stimulation of Ia afferents is not sufficient to reduce deficits in pre- and postsynaptic components of glutamatergic and cholinergic signaling, and disclose that MNs after SCT do not transcribe the perceived cues into compensatory response at the molecular level, when supported with proprioceptive stimuli at the early postlesion period.

## Materials and methods

### Animals

The experiments were carried out on 37 adult male Wistar rats, weighting 340–360 g at the beginning of the experiment. Animals were outbred colony (International Laboratory Code Registry: Cmdb:Wi) of the Wistar rats supplied by Medical University of Białystok, Poland.

The animals were bred in the animal house at the Nencki Institute of Experimental Biology in Warsaw, Poland. They were given free access to water and pellet food and were housed under standard humidity and temperature conditions on a 12 h light/dark cycle. Rats were initially housed in groups of 4–6 but after surgery they were individually housed.

Experimental protocols involving animals, their surgery and care were approved by the First Local Ethics Committee in Warsaw (no 782/2015), in compliance with the guidelines of the European Community Council Directive 2010/63/UE of 22 September 2010 on the protection of animals used for scientific purposes. Animals were divided into two groups: Control (CN), with no electrodes implanted (7 rats used for immunohistochemical study, 9 rats used for gene expression study), and Experimental (6 rats used for immunohistochemical study, 6 rats used for gene expression study), subjected to bilateral implantation of electrodes into Sol muscles and nerve cuff electrodes over the tibial nerves and subsequent complete transection of the spinal cord (SCT) at low-thoracic segments (Th9/10) (see Table 1). Both groups of rats received bilateral intramuscular tracer injections to the LG and TA. Additional experimental group of 9 rats subjected to SCT and neuro-tracing, but not implanted, was prepared and compared with SCT-implanted group to determine the effect of SCT itself and of electrodes implantations on gene expression in MNs. Unilateral electrical stimulation of low-threshold muscle afferents in the tibial nerve was controlled by means of H-reflex recorded in the Sol muscle [9, 10].

**Table 1:** The number of rats and of labeled α-motoneurons (MNs) in the groups in which measurements of density and volume of VGLUT1 IF and VAChT IF boutons abutting on the MN cell bodies and their proximal dendrites were done.

	Gastrocnemius Lateralis (LG) MNs		Tibialis Anterior (TA) MNs	
Animal Group	Number of rats	Number of MNs	Number of rats	Number of MNs
Control (CN)	7	49	7	39
Sham (SCT-NS)	6*	27	4^	14
Stimulated (SCT-S)	6*	28	4^	15

Out of 7 SCT animals participating in the unilateral, low-threshold stimulation of the tibial nerve, one animal was rejected from the analysis of the Sham side due to the damage of electrodes and from the analysis of the Stimulated side due to incorrect electrode implantation (*). In two animals the number of labeled TA MNs was low (^) therefore they were not taken to analysis.

### Retrograde labeling of motoneurons, implantation of electrodes and post-surgery care

The intramuscular injections of tracers to label MNs and implantation of the electrodes were performed during the same surgery session in the surgery room, under aseptic conditions. The animals were given subcutaneous injection of Butomidor (Butorfanolum, 1.5 mg/300 g b.w.; Richter Pharma, Wels, Austria) as a premedication and then were anesthetized with isoflurane (1–2.5% in oxygen; Aerrane, Baxter, USA, Lessines, Belgium) via a facemask. The skin overlying LG muscle was shaved, disinfected with 3% hydrogen peroxide, and cut. For immunohistochemical study, fluorescence neurotracers: cholera toxin conjugated with Alexa Fluor 555 (0.01% aqueous solution, Molecular Probes, USA) and Fast Blue (1% aqueous solution, Dr. Illing Plastics GmbH, Germany) were injected to LG and TA muscle, respectively, by means of Hamilton microsyringe with 22-gauge needle attached. For gene expression study in microdissected MNs, cholera toxin conjugated with Alexa Fluor 555 (0.01% solution in phosphate buffered saline, Molecular Probes, USA) and cholera toxin conjugated with Alexa Fluor 488 (0.01% solution in phosphate buffered saline, Molecular Probes, USA) were injected to LG and TA muscle, respectively, in the same way. The needle was gradually advanced from the distal toward the proximal end of the muscle belly. After 5 min delivery of the tracer the needle was left in the muscle for at least 3 min in order to avoid leakage of the dye and then slowly withdrawn from the muscle belly. The injection site was cleaned with warm 0.9% NaCl solution after retraction of the needle and the skin was sutured.

Procedures and the surgery necessary for implantation of the electrodes were the same as those described in detail in our former article [10]. Briefly, a connector plug for the electrodes was sewn to the muscles and ligaments over the vertebrae. The electrodes were drawn subcutaneously, bilaterally, from the connector plug mounted at the back, to the Sol muscles and the tibial nerves to be implanted. Bipolar cuff electrodes were performed as described by Loeb and Gans [24] and implanted bilaterally over the tibial nerves which were separated from the common peroneal and sural branches in the popliteal fossae. Unilateral low-threshold stimulation of the tibial nerve produced a compound muscle action potential, which was recorded in the Sol muscle by means of a pair of Teflon-coated fine-wire electrodes with 1.5 mm final bare spaced apart by approximately 5 mm. To avoid leakage of the dye from the Sol muscles with electrodes implanted, the dye was injected to the LG muscles, a close synergist of Sol, which are also innervated by tibial nerve branches and thus subjected to electrical stimulation via cuff electrode.

After the surgery, Baytril (Enrofloxacinum 5 mg/kg; Bayer GmbH, Leverkusen, Germany) was administered over five consecutive days to prevent infection. An analgesic Tolfedine (Tolfenamic acid 4%, 4 mg/kg, s.c., Vetoquinol S.A., Lure Cedex, France) was given during the first five postoperative days. After the surgery, the rats were placed in warm cages and inspected until fully awaken. Thereafter plastic collars (Harvard Apparatus, Holliston, Massachusetts, USA) were put on each animal to protect their wounds from licking, and rats were returned to individual cages with access to food and water *ad libitum*.

### Spinal cord transection and postoperative care

Two weeks after first surgery a complete transection of the spinal cord was performed in the surgery room, under aseptic conditions. The animals were anesthetized as described above. Caudal thoracic vertebrae were exposed by incision of the skin and muscles with a fine scalpel. A laminectomy was performed at the level of T9/10 vertebrae. The dura was opened and Lignocainum hydrochloricum (2%, Polfa Warszawa S.A., Poland) was applied to the surface of the cord before the spinal cord was completely transected using surgical scissors. The gap between the rostral and caudal ends of the spinal cord was enlarged by aspiration to about 0.5 mm, washed with warm (approximately 36°C) 0.9% NaCl solution and dried with absorbable cellulose. After careful inspection of the lesion area by means of surgical microscope (SEILER COLPOSCOPE 955 Seiler Precision Microscopes, St. Louis MO, USA) the surrounding tissues and skin were closed.

After the surgery about 5 ml of 0.9% NaCl solution was injected subcutaneously. The antibiotic Sultridin (30 mg/kg, Norbrook, Ireland) was administered during five consecutive days and an analgesic Vetaflunix (2.5 mg/kg, subcut., VET AGRO, Poland) for three postoperative days. Immediately after the surgery, the rats were placed in warm cages, covered with blankets and inspected until they recovered after anesthesia. Thereafter they returned to individual cages with access to food and water *ad libitum*. Three times daily during the first postoperative week and twice daily in the subsequent week, the animals were attended for general inspection, cleaning of their body and, if necessary, for manual bladder expression. The animals had no significant health problems for the whole period of experiment except for occasional bladder bleeding during initial postsurgery days. Spontaneous micturition usually returned in the second post-surgery week.

### Behavioral training

To reduce the effects of behavioral context and ongoing motor activity on the recorded H-reflexes the animals were accustomed to being restrained in the apparatus which limited their body movements. Therefore, approximately two weeks before the stimulation experiment was initiated, the animals started to be accustomed to sitting in the restraining apparatus until they reached 20 min criterion [10]. The hindlimbs of the spinal animals were positioned in the apparatus on the plantar aspect of the feet, as in the CN group, and corrected if necessary.

### Low-threshold proprioceptive stimulation of the tibial nerve and Hoffmann reflex recording

The monosynaptic Hoffmann reflex (H-reflex) was elicited by electrical stimulation of low-threshold muscle afferents (group Ia) in the tibial nerve and recorded as a compound muscle action potential in the Sol muscle as described in detail in our former article [10]. Briefly, the tibial nerve was stimulated unilaterally (SCT-S), the contralateral side served as a sham-stimulated (SCT-NS). Continuous bursts of three pulses (pulse width = 200 μs with 4 ms inter-pulse interval) were delivered every 25 ms to the tibial nerve in four 20 min sessions daily for seven consecutive days [25]. After each session the animals rested at least 1 hour in their home cages and were rewarded with corn cookie. On the day preceding SCT the current threshold eliciting the H-reflexes and/or M-responses was established for every animal and the responses to 30 single pulses of 500μs duration delivered at 0.3 Hz were recorded. This control procedure was repeated daily during seven days of stimulation. The strength of stimulation, established during daily control sessions, was near the threshold of excitation of the motor fibers, which is higher than the one activating Ia afferents. This current elicited a fair H-reflex, since the majority of Ia fibers were already excited when the direct motor response (M) was at its threshold [26].

During stimulation with continuous bursts of three pulses only the M-responses (R1) elicited by the first out of three pulses were not contaminated by subsequent H-reflexes and these were strictly controlled and kept near its threshold. The 4 ms time intervals between consecutive pulses in the burst allowed to observe the recruitment of R2 and R3 responses classified as composed of M2 + H1, and M3 + H2, respectively. The R4 response to the third stimulus, appearing with the latency of approximately 6 ms, was classified as the H-reflex.

### Data acquisition and analysis of electrophysiological data

Acquisition and analysis of electrophysiological data have been described in detail in our former article [10]. Briefly, after amplification the analog EMG signals were fed to a CED Micro 1401^mk II^ interface (Cambridge Electronic Design Ltd, UK), digitized and fed to a PC. Raw EMG activity was also monitored throughout the experiment on the oscilloscopes. A Spike 2 (Cambridge Electronic Design Ltd, UK)-based script was used to measure the latency, peak-to-peak amplitude and the area of the H-reflex and M-response and to average these data. These results were expressed as a percentage of M_max_ elicited by single-pulse stimuli and collected in each animal before SCT and after the last session on the 6^th^ day of stimulation.

### Tissue processing for immunohistochemistry

Within 1.5 h after the last stimulation session, the animals were anaesthetized with lethal dose of Morbital (pentobarbital 120 mg / kg body weight, intraperitoneal; Biowet Puławy Sp. z o.o., Poland) and perfused transcardially with 250 ml 0.01M phosphate-buffered saline (PBS), pH 7.4, at room temperature (25°C; RT) and, subsequently, with 550 ml ice-cold fixative (4% paraformaldehyde, PFA, in 0.1 M phosphate buffer (PB)). Spinal cords were removed from the vertebral columns and were post-fixed in the same fixative for 1 hour at 4°C. Tissue was then cryoprotected stepwise in 10%, 20% and 30% sucrose in 0.1M PB at 4°C and stored at 4°C until use. The L1-L5 spinal cord segments were surrounded by Jung tissue-freezing medium (Leica, Nussloch, Germany, cat no 14020108926) and frozen in cryostat at -70°C. After tissue blocks were placed on tissue holders, the sham-stimulated side was marked with thin needle. Transverse 25μm thick sections were cut on the cryostat at -20°C, collected free-floating in anti-freeze cryoprotectant solution (300 ml glycerol, 500 ml 0.05 M PB pH 7.4, supplemented with 150 g sucrose and 90 mg thimerosal (Sigma-Aldrich, St. Louis, MO, USA; cat no T5125)) and stored at -18°C until further processing.

### Immunofluorescence labeling of synaptophysin and vesicular glutamatergic (VGluT1) and cholinergic (VChAT) transporters

Immunofluorescence (IF) triple-labeling was carried out in one experimental session to assure identical conditions of tissue processing and staining. The selected free-floating sections with traced MNs (6–14 sections per animal; their number was limited by the strength of the tracer’ IF signal in MNs, credible to identify their perikarya) were washed in PBST (PBS +0.2% Triton X-100 (Sigma-Aldrich, St. Louis, MO, USA, cat no X100)) and incubated with a solution of 2.5% normal goat serum (NGS; PAA Laboratories, Linz, Austria) and 2.5% normal donkey serum (NDS; Abcam, Cambridge, UK, cat no ab7475) in PBST for 30 min at RT, in order to prevent them from the non-specific staining. Next, sections were incubated overnight at 4°C with rabbit anti-VAChT (1:1000, Merck-Millipore, USA, cat no V5387) combined with mouse anti-VGLUT1 (1:500, Synaptic Systems GmbH, Germany, cat no 135 511) and guinea pig anti-synaptophysin antibodies (1:1000, Synaptic Systems GmbH, Germany, cat no 101 004), diluted with PBST. Next, sections were washed in PBST, prior to 1h incubation at RT with the respective secondary antibodies: Alexa Fluor 647 donkey anti-mouse (1:400, Jackson Immuno-Research Lab Inc., USA, cat no 715-606-150), Alexa Fluor 568 goat anti-rabbit (1:500, cat no A-11036) and Alexa Fluor 488 goat anti-guinea pig (1:500, Invitrogen, USA, cat. no A-11073). After several washes, the sections were put on the glass slides, air-dried, mounted in Mowiol medium containing 0.1 M Tris-HCl buffer (pH 8.5), 20% glycerol, 10% Mowiol (Sigma-Aldrich, St. Louis, MO, USA, cat no 81381), and 2.5% 1,4-diazabicyclo [2,2,2] octane (Sigma-Aldrich, St. Louis, MO, USA, cat no D2522), cover-slipped and stored in the dark at 4°C until analysis. The regular staining procedure was preceded by the assay comparing single and triple labeling to test whether simultaneous triple reaction does not impair or cause non-specific antibody binding. Negative controls obtained by omitting the primary antibodies provided no staining.

### Image acquisition, deconvolution and selection criteria for quantification

The immunolabeled, retrogradely traced MNs were captured with confocal inverted microscope LSM 780 (Carl Zeiss, Jena, Germany). Z-stacks of images of labeled MNs and terminals apposing their perikarya consisted of digital slices collected at 0.21 μm intervals with a pixel size of 0.065, using PL APO 40x (1.4 NA) DIC oil-immersion objective. Images were collected at constant exposure parameters for each of four channels detecting Fast Blue or Cholera toxin IF tracers, and IF signal for VGLUT1, VAChT and synaptophysin.

The number of examined LG and TA MNs and the number of VGLUT1 IF and VAChT IF boutons abutting on these MNs are shown in the Table 1.

Z-stacks of images were subjected to deconvolution procedure using Huygens Professional software (Scientific Volume Imaging, Hilversum, Netherlands) to reduce the image distortion arising from light scattering. Based on random set of images an experimentally blind rater determined parameters of the deconvolution algorithm to yield maximal resolution for each antibody. The level of background and signal to noise ratio was selected for each channel independently.

**Fig 1:**
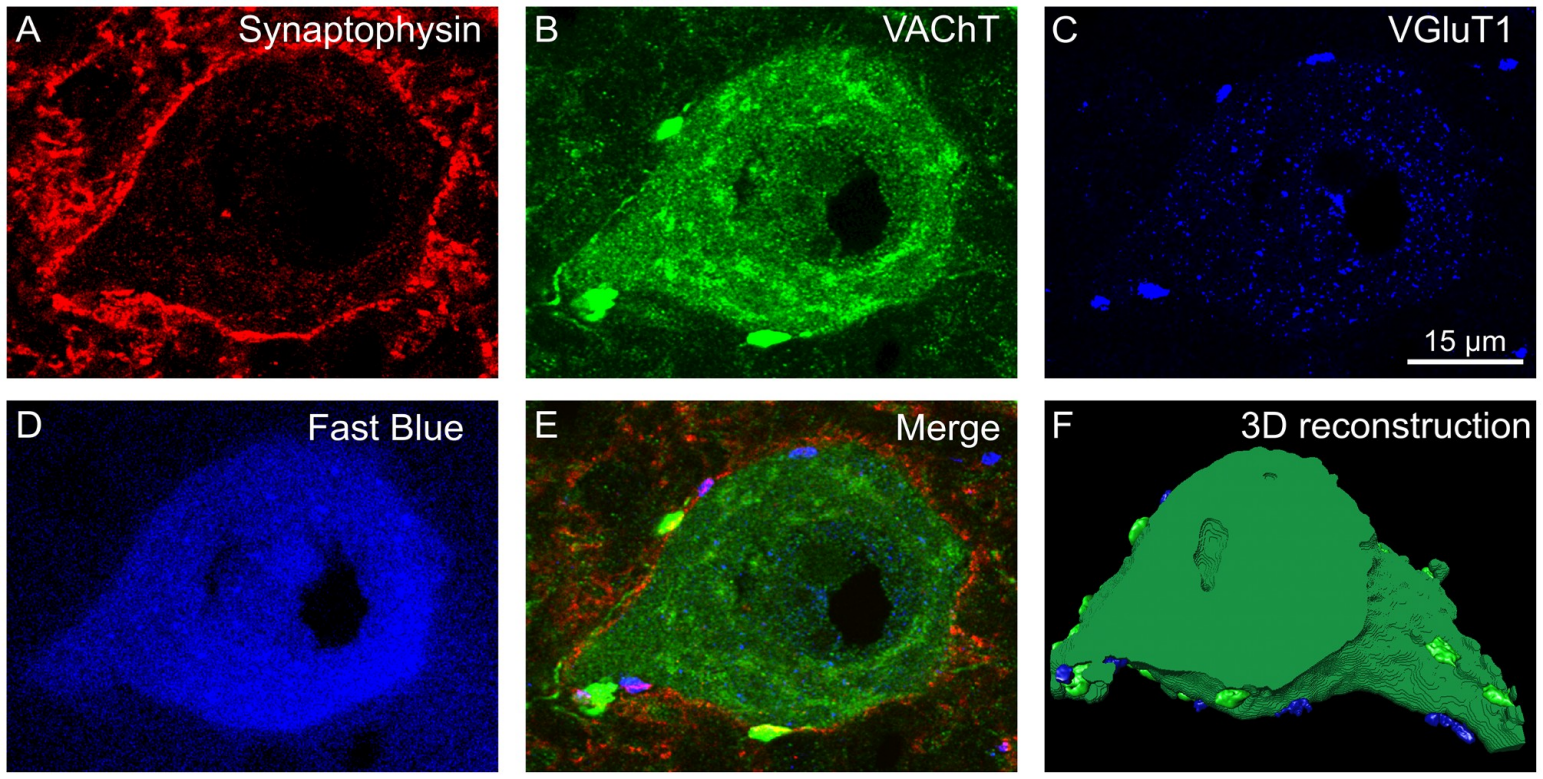
Three-dimensional reconstruction of LG α-motoneuron labeled with Fast Blue (FB) tracer injected into the LG muscle of the control animal. A-D. Single optical section (#8 out of collected 55) showing synaptophysin labeling of terminals (A) in the proximity of α-motoneuron identified with FB (D), overlapping with VAChT IF C-boutons (B) and VGluT1 IF Ia boutons (C) shown on merged A-C images (E). F. The same neuron reconstructed from 55 optical sections for the measurement purposes, showing lateral surface of perikaryon and two protruding dendrites, with the masks of boutons (VAChT IF—green, VGluT1 IF—blue; pseudocolors).

While the presence of apparent appositions at the LM level does not ensure synaptic contact, we made an assumption of a contiguity of glutamatergic and cholinergic boutons to the cell body or proximal part of a dendrite, when identified with VAChT cytoplasmic staining and verified with bouton overlap with synaptophysin, examined on single optical sections (see triple labeling of a single optical section and reconstruction of 55 scans of the LG MN, Fig 1). The same criteria were used in our previous papers [1, 9]. A segment of a dendrite within 50 μm from the soma has been taken as a proximal dendrite, after a classical study of Brannstrom [27], who showed, that a similar number of boutons contact the soma and proximal dendrites in FF, FR and S MNs, in contrast to dendritic segments located more distally.

The Z-stack of digital images allowed for 3D reconstruction (voxel size (X/Y/Z) of 0.065 x 0.065 x 0.21 μm^3^) and analysis of 30–50% of the MN soma surface, done by means of ImagePro Premiere software (version 9.1, Media Cybernetics, Inc., Rockville, USA). Created for our purpose macro was used for semiautomatic measurement of the number and volume of individual IF-synaptic terminals on the surface of MNs.

To render a detailed surface of MNs, their 3D masks were created based on IF signal of VAChT detected in the MN cytoplasm and of synaptophysin signal adjacent to the plasma membrane (see Fig 1A and 1B). The threshold values were automatically determined and verified by experimenter providing the best fit. The 3D mask reflecting the whole VAChT IF signal detected in the field was reduced to the 3D mask of MN soma, shown for the reconstructed cell fragment in Fig 1F. The space occupied by the cell nucleus, devoid of VAChT IF, was artificially filled. From this selection, after subtracting the top and bottom surfaces, the area of the lateral surface of the cell body fragment was calculated.

The mean of the lateral surface of analyzed fragments of the MNs soma was balanced between groups and amounted to 4324 +/- 2018 μm^2^ (mean +/-SD). In order to normalize the results, the density of synaptic boutons was expressed as their number abutted on 3000 μm^2^ of the lateral surface of the soma, i.e. the same fragment of the MNs soma, which we referred to in our previous study [1]. To create a detailed, three-dimensional surface rendering of the IF signal of VAChT and VGluT1-immunolabeled synaptic terminals, we used automatic thresholding functions of the software. The intensity of IF of labeled vesicular transporters was much stronger within synaptic terminals than that in the cytoplasm of MN perikarya (see Fig 1B and 1C) enabling unequivocal discrimination of those terminals. Only objects apposing cell surface were saved for the analysis. The criteria for the volume of glutamatergic terminals (objects larger than 1 μm^3^) and cholinergic terminals (objects larger than 5 μm^3^) for the intact animals were adopted from other studies [28–30] and used for VAChT measurements in our previous studies [1]. The numerical data were exported to Excel for further analysis. The figures were assembled using COREL DRAW X5 software.

### Tissue preparation and isolation of motoneurons by means of laser microdissection

Rats were deeply anaesthetized with a lethal dose of Morbital (Biowet Puławy, Poland) and perfused transcardially with ice-cold saline. Lumbar spinal cord L3-6 segments from CN (N = 9), SCT (N = 9), and SCT + electrode-implanted (N = 6) rats were dissected immediately in the cold room, frozen on dry ice and stored at -80°C. The spinal cord segments were surrounded by Jung tissue-freezing medium (Leica, Nussloch, Germany, cat no 14020108926) and cut into 20 or 25 μm thick longitudinal sections on the cryostat (Leica CM1850) at −20°C. Sections were mounted onto RNase-free PET-Membrane Frame Slides (Leica, cat. no 11505190) and immediately placed in dry ice-precooled box. Slides were stored at -80°C until processing (up to several weeks). Prior to laser miscrodissection (LMD), sections were dehydrated stepwise in 70%, 80%, 90%, 2x 100%, ethanol for 30s each, submerged in 2x 100% xylene to remove ethanol (30s for the first time; 3 min for the second time) and air dried for 3–5 min. Leica Laser Microdissection system LMD7000 was used to isolate LG and TA MNs labeled with neurotracers. Single slides with dehydrated sections were placed on the holder of the LMD system and RNase-free caps of 0.2 ml tubes filled with 20 μl extraction buffer (from Arcturus^™^ PicoPure^™^ RNA Isolation Kit, KIT0204, Applied Biosystems) were placed in the tube holder for sample collection. By means of LMD system microscope (objectives x10, 0.32 NA and x63, 0.7 NA) attached to the software unit, MNs were identified, selected and then cut with UV laser. The dissected sections of MNs dropped via gravity into prepared tube cap. To limit RNA degradation, samples were collected for up to 60 min per slide. Collected MNs were incubated with extraction buffer for 30 min at 42°C to lyse the cells; lysates were centrifuged at 800 x g for 2 min. Then samples were immediately frozen on dry ice and stored at −80°C.

### Tissue processing for Real-Time qRT-PCR

The total RNA from LG and TA MNs was isolated by using Arcturus PicoPure^™^ RNA Isolation Kit (KIT0204, Applied Biosystems) according to the manufacturer’s protocol, followed by RNA pre-amplification with the use of QuantiTect^®^ Whole Transcriptome kit (Qiagen, cat no. 207043, 207045) to synthesize the cDNA. The final concentration of the cDNA was determined by utilizing Quant-iT^™^ PicoGreen^™^ dsDNA Assay Kit (Thermo Fisher Scientific, cat no. P7589). Gene expression level was analyzed by RT-qPCR with glyceraldehyde-3-phosphate dehydrogenase (GAPDH) as internal control gene. The RT-qPCR reactions were performed in a 20 μl reaction mixture containing 7.4 μl PCR grade H_2_0, 10 μl LightCycler^®^ 480 Probes Master solution (Roche, cat no. 04887301001), 0.4 μl forward primer (20μM) of target gene, 0.4 μl reverse primer (20 μM) of target gene, 0.4 μl probe (10 μM) of target gene, 0.1 μl forward primer (20 μM) of GAPDH gene, 0.1 μl reverse primer (20 μM) of GAPDH gene, 0.2 μl probe (10 μM) of GAPDH gene, 1 μl cDNA (diluted 20 times according to the concentration). Roche LightCycler^®^ 96 Instrument (Roche Applied Science, Penzberg, Germany) was used to run the reactions with the following thermal cycling profile: preincubation at 95°C for 10 minutes, 60 cycles of denaturation at 95°C for 10 seconds, annealing at 60°C for 10 seconds, and extension at 72°C for 10 seconds. Data were analyzed by using the LightCycler software. The 2^-ΔΔCt^ method has been used as a relative quantification strategy for data analysis based on the target and reference genes’ Ct (cycle threshold) value, the cycle number at which the amplification curve reaches the threshold line.

Target-specific probes, forward and reverse primers designed by Universal Probe Library Assay Design Center were used (Table 2).

**Table 2:** List of probes and primers.

Gene symbol	Gene name	Accession number	Probe number (Roche UPL)	Amplicon length/nt	Forward primer (5’–3’)	Reverse Primer (5’–3’)
Gria2	AMPA receptor GluR2 subunit	NM_017261.2; NM_001083811.1	67	77	ggaggtgattccaaggaaaag	Cccccgacaaggatgtaga
Grin1	NMDA receptor NR1 subunit	U11418.1	95	94	cctacacagctggcttctacag	Cgaaggaaactcaggtggat
Chrm2	Muscarinic receptor M2	NM_031016.1	64	76	ccaccttcagactgtcaacaatta	catggagaaaacacctatgatgag
Gapdh	Glyceraldehyde-3-phosphate dehydrogenase (GAPDH)	NM_017008.4	*	92	Ctgcaccaccaactgcttag	Tgatggcatggactgtgg

*sequence: tttggcatcgtg. Synthesized by Institute of Biochemistry and Biophysics, Polish Academy of Sciences, Warsaw, Poland

### Statistical analysis

The Shapiro-Wilk test was used to verify normality of data distribution. The homogeneity of variance in the groups was analyzed with the Leven test. Because normality of data distribution was not met in some experimental groups, the non-parametric Wilcoxon and Mann-Whitney U tests were used for comparisons of related samples and independent samples, respectively. STATISTICA 13 software (StatSoft. Inc. Tulsa, OK, USA) was used for the data analysis.

## Results

### Effects of SCT and stimulation of low-threshold proprioceptive afferents in the tibial nerve on the H-reflex in the soleus muscle

**Fig 2:**
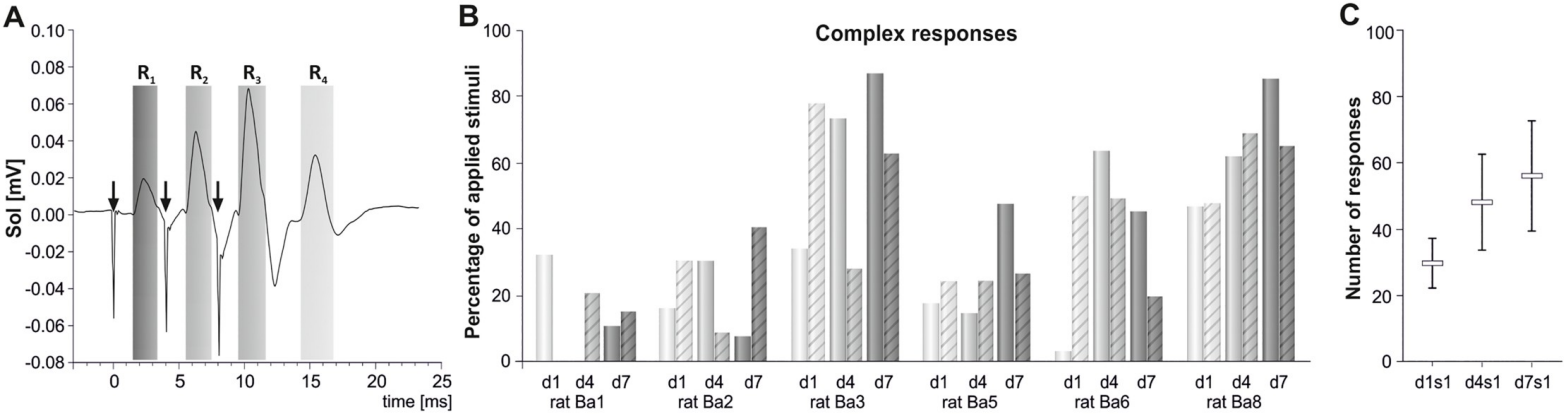
The effect of 7 days of stimulation with the continuous burst stimuli on the complex responses recorded in the soleus muscle of the spinal rat. **A**. The responses to 9600 bursts stimuli were averaged. To keep control over the current sufficient to elicit the fair H-reflex the stimulation current was adjusted at the threshold necessary for eliciting M responses. The direct motor responses (R1), complex R2 and R3, and H-reflex (R4) are shown. Arrows point to the stimulus artifacts. **B**. The frequency of eliciting complex responses to continuous burst stimulation of the tibial nerve collected during the first (d1), fourth (d4) and last (d7) days of stimulation in the first (plain bars) and the last (dashed bars) daily sessions in six spinal rats. Each bar represents the percentage of complex responses elicited by 118 bursts of stimuli. **C**. The mean number of complex responses to continuous burst stimulation of the tibial nerve collected during the first (s1) daily sessions on the first (d1), fourth (d4) and last (d7) days of stimulation. These responses are shown as means (+/- SEM) from 6 rats.

**Fig 3:**
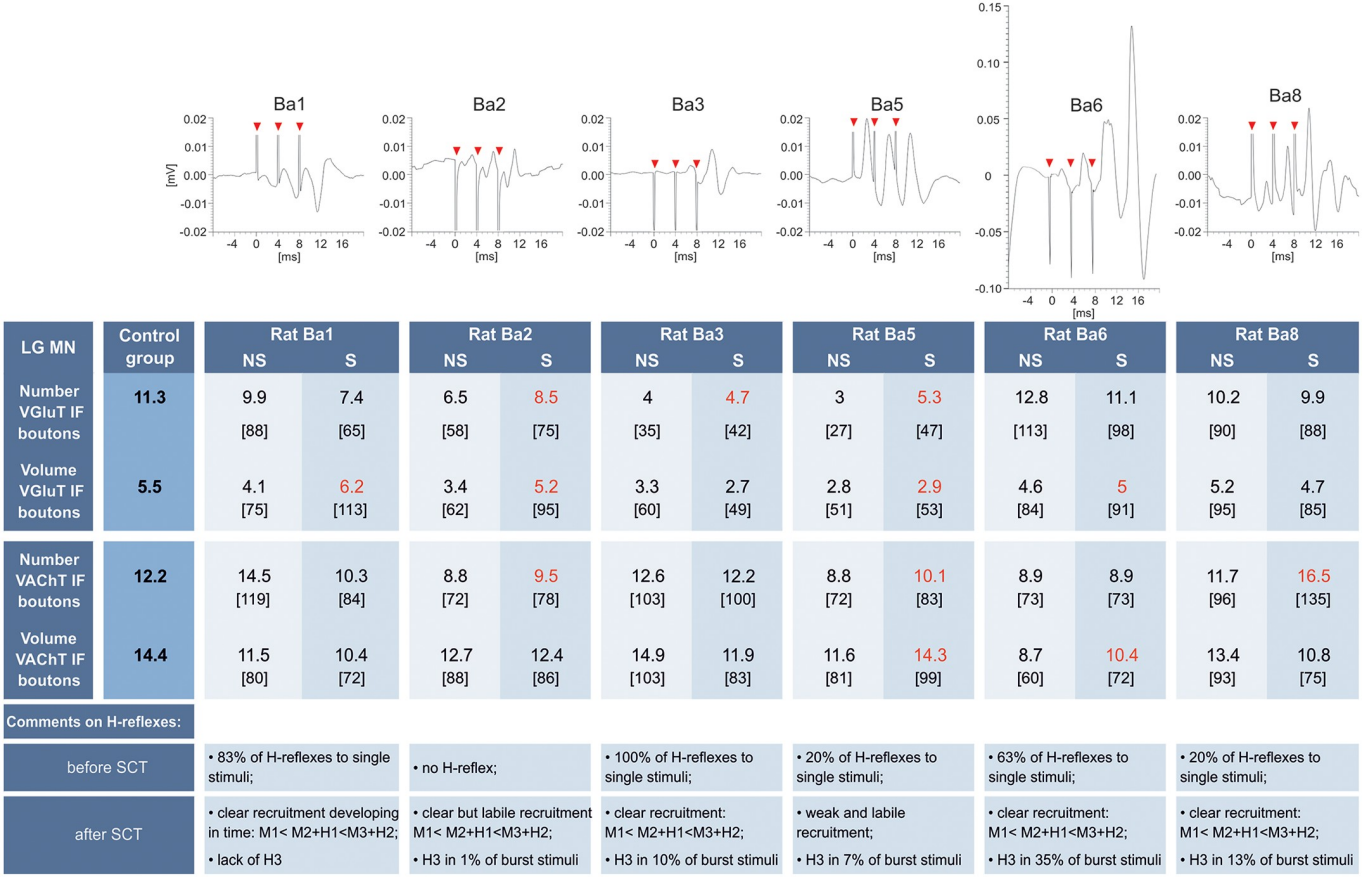
Individual changes in the number and volume of VGLUT1 IF and VAChT IF boutons abutting LG MNs together with the averaged H- and M responses collected in the last (7th) day of stimulation nine days after SCT. From the top: averaged responses to 9 600 burst stimuli showing the recruitment of complex (R2 and R3) responses. Additional information on the H-reflexes in every animal is at the bottom. Middle: mean number and volume of VGLUT1 IF and VAChT IF boutons in the intact animals (CN) and in the stimulated (SCT-S) and sham-stimulated (SCT-NS) animals after spinal cord transection (SCT). The density of synaptic boutons is expressed as their number abutted on 3000 μm^2^ of the lateral surface of the soma. Percentages of control values are shown in brackets. In red: values indicating an increase of the number and/or volume of VGLUT1 IF and VAChT IF boutons on the S comparing to NS side. Seven CN rats and six rats with SCT subjected to unilateral, electric stimulation, were used for these analyses.

Before SCT the control H-reflexes tested with single pulses delivered at 0.3 Hz were observed in all but one animals. The current set at the threshold eliciting M-responses was optimal for eliciting H-reflexes. The latencies of H-reflexes and M-responses in the soleus muscle were approximately 6 ms and 1.5–2 ms, respectively. Two days after SCT we could not elicit the regular H-reflexes to single pulses in four of six animals. However, we observed the recruitment of the complex R2 and R3 responses (see Fig 2A) after stimulation with continuous bursts of 3 pulses, indicating that a fraction of low-threshold proprioceptive fibers was successfully activated in the H-reflex loop. The probability that the current was below the threshold of M-responses was higher than that of exceeding the threshold; the frequency of the M1 responses was on average 10% (see S1 Table). This paradigm of stimulation allowed to control and analyze the amplitude of the direct motor responses (R1 = M1) as well as of complex R2 and R3, and H-reflex (R4) (Figs 2 and 3). Fig 3 shows individual results collected in all animals during the last day of stimulation after SCT. Multiple overlapping traces from numerous bouts of stimulation showing variability of response in awake animals are exemplified on S1 Fig.

Fig 2B and 2C show cumulative effects of daily sessions and of consecutive days of stimulation on a frequency of complex responses. The cumulative effect of daily sessions was observed in all but one animal during the first day of stimulation only. In the next days it diminished, and reversed in 4 out of 6 animals in the last stimulation day, when the frequency of complex responses was higher during the first than the last daily sessions. It indicates daily reduction in the excitability of the H-reflex loop. Fig 2C shows enhancing effect of 7-days stimulation on frequency of the complex responses (compare d1s1 vs d4s1 vs d7s1). Altogether, the presented results of increased frequency of the complex responses to the burst stimulation of the tibial nerve indicate that H-reflex loop was effectively activated during stimulation and its effectiveness tended to increase in consecutive days of stimulation.

### Does stimulation of low-threshold proprioceptive afferents enrich glutamatergic and cholinergic innervation of LG α-motoneurons in spinal rats

**Fig 4:**
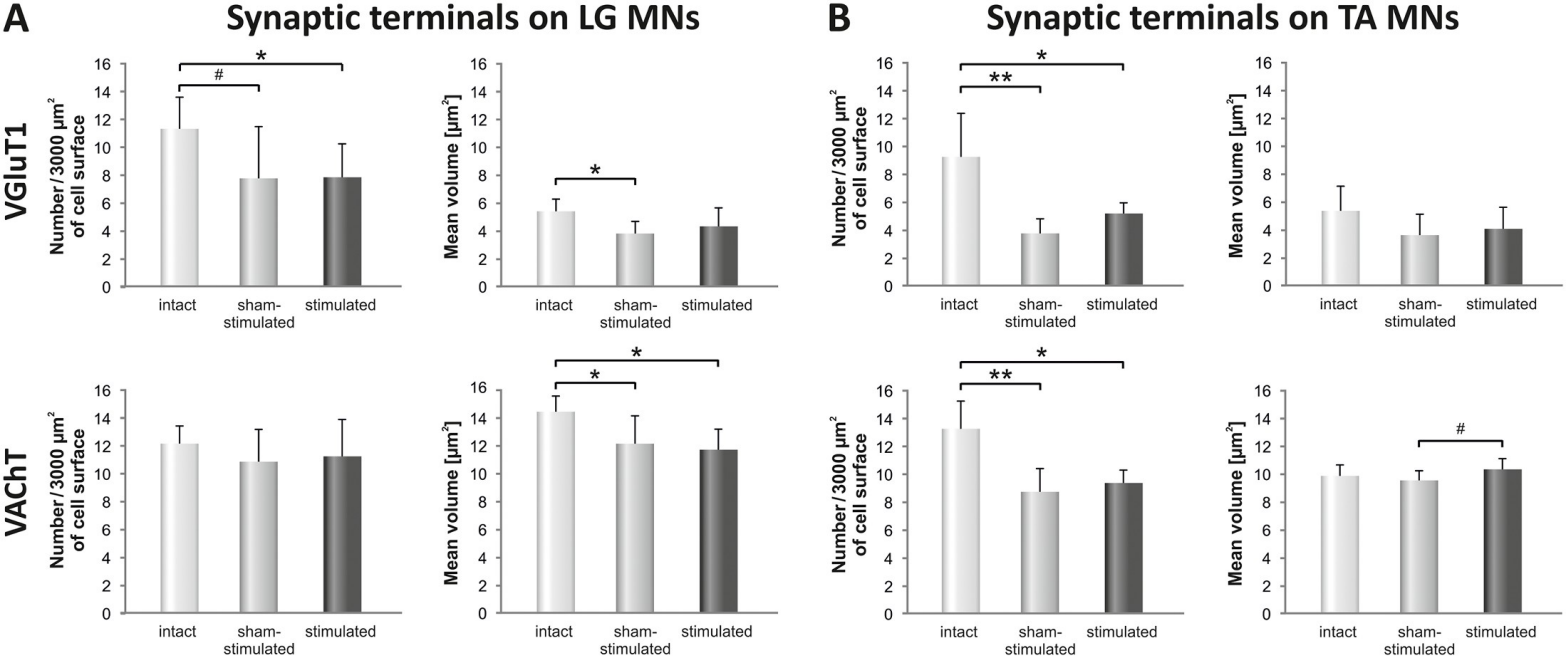
Comparison of changes in the number and volume of VGLUT1 IF and VAChT IF boutons abutting LG (A) and TA (B) MNs. The effect of SCT, followed by seven days of stimulation of the group I proprioceptive fibers in the tibial nerve is shown. Data are means +/- SD from 7 Control, 6 (LG MNs) and 4 (TA MNs) SCT and stimulated rats. **p < 0.01, *p<0.05, ^#^p = 0.086, Mann-Whitney U test. In comparisons between sham-stimulated and stimulated sides ^#^p = 0.067, Wilcoxon test.

To reveal the effects of SCT on glutamatergic Ia input and cholinergic V0c input to LG MNs, the density and volume of VGLUT1 IF and VAChT IF boutons apposing immunolabeled MNs on the sham-stimulated side of the spinalized rats were compared with those in the non-transected, CN group (Figs 3 and 4). The density of synaptic boutons is expressed as their number abutted on 3000 μm^2^ of the lateral surface of the soma. In CN rats the mean density of VGluT1 IF boutons apposing LG MNs was 11.3 +/- 2.46 (+/- SD) and was non-significantly higher than the density of VGluT1 IF boutons on TA MNs, which amounted to 9.2 +/- 3.26. The mean density of VAChT IF boutons apposing LG and TA MNs was similar, amounting to 12.2 +/- 1.37 and 13.2 +/- 2.02 terminals, respectively.

In the analysis of the effects of stimulation on these inputs, we compared individual neuroanatomical data with averaged complex R2 and R3 responses collected during the last day of stimulation, to search for the relationship between the degree of input impairment and stimulation effectiveness (Fig 3).

The first conclusion emerging from that analysis is a clear differentiation in responses of glutamatergic innervation of LG MNs to SCT. Although in all but one animal the number of VGLUT 1 IF terminals abutting LG MNs decreased after SCT, in half of the group the effect was profound (rats 2, 3, 5) while in the other half (rats 1, 6, 8) it did not exceed 25% (Fig 3). In the latter group the stimulation of proprioceptive fibers slightly reduced the number of VGLUT 1 IF boutons comparing to the non-stimulated side whereas in the group with deeply reduced number of VGLUT1 IF, the stimulation partly compensated their loss.

The second conclusion is that 9 days after SCT the cholinergic innervation of LG MNs was less affected comparing to the group Ia proprioceptive glutamatergic innervation; its loss did not exceed 28% of control values (e.g. in rats 2 and 5). On the stimulated side the number of VAChT IF terminals was slightly higher (rats 2 and 5) or did not change (Fig 3).

The effect of SCT on the volume of VGLUT 1 IF boutons was also stronger in the group composed of rats 2, 3, 5 than those of 1, 6, 8 but the changes induced by stimulation were inconsistent in these subgroups. There was a decrease of the volume of VAChT IF boutons after SCT and an inconsistency in their response to stimulation (Figs 3 and 4).

Fig 4 shows that the mean density of VGLUT1 IF boutons contacting perikarya of LG MN was decreased by 32% after SCT (p = 0.086, Mann-Whitney U test). Their mean volume was reduced by 29% (p = 0.022, Mann-Whitney U test). Changes in VAChT IF boutons apposing LG MNs after SCT were smaller comparing to those in VGLUT1; their mean density tended to decrease by 11% (p = 0.25, Mann-Whitney U test) whereas their mean volume decreased by 16% (p = 0.045, Mann-Whitney U test).

Seven days of low-threshold stimulation of proprioceptive afferents in the tibial nerve hadn’t change the mean density, neither had it change the mean volume of VGLUT1 IF (p = 0.50 and p = 0.46, respectively, Wilcoxon test) and VAChT IF (p = 0.92 and p = 0.60, respectively, Wilcoxon test) boutons abutting LG MNs, when compared with the results in the sham-stimulated side (Fig 4).

**Fig 5:**
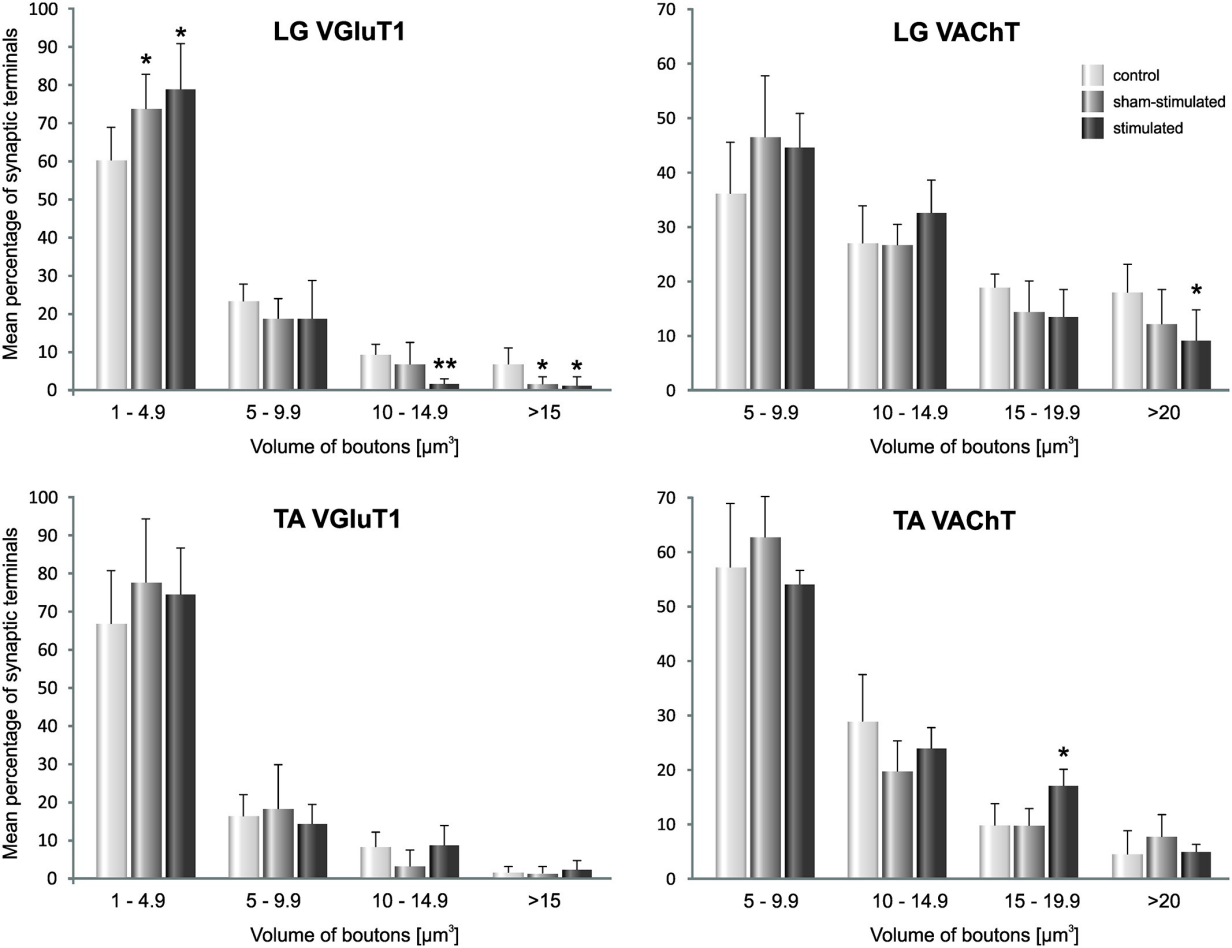
Changes in volume of VGLUT1 IF and VAChT IF sized boutons abutting LG and TA MNs. The effect of SCT, followed by seven days of stimulation of the group I proprioceptive fibers in the tibial nerve is shown. Four volume classes of boutons were distinguished. Note that after SCT and stimulation the number of the smallest VGLUT1 IF boutons in LG increased while the bigger ones decreased. The same tendency occurred in the population of VAChT boutons abutting LG MNs. Data are means +/- SD from 7 Control, 6 (LG MNs) and 4 (TA MNs) SCT and stimulated rats.**p < 0.*01*, *p<0.*05*, *Mann-Whitney U test*.

To examine the possibility that the volume of particular subpopulations of terminals was increased by stimulation, a detailed analysis of changes in volume of sized VGLUT1 IF and VAChT boutons on LG MNs was carried out. The analysis showed that both after SCT and stimulation the number of the smallest VGLUT1 IF boutons increased, while the number of the bigger ones decreased. The same pattern of changes was observed in size-distribution of VAChT IF boutons (Fig 5).

### Changes in glutamatergic and cholinergic innervation of TA α-motoneurons in spinal rats

The effect of SCT on the density of VGLUT1 IF and VAChT IF boutons apposing TA MNs was more pronounced than on LG MNs (Fig 4). The density of VGLUT1 IF boutons was reduced by 60% after spinalization (p = 0.008, Mann-Whitney U-test), and the volume of the preserved boutons tended to decrease (by 33%; p = 0.19, Mann-Whitney U-test).

The density of VAChT IF boutons was decreased by 34% after spinalization (p = 0.008, Mann-Whitney U-test). The mean volume of VAChT IF boutons apposing TA MNs was not affected by SCT (p = 0.45, Mann-Whitney U-test).

Stimulation did not affect the density of VGluT1 IF and VAChT IF boutons apposing TA MNs. A 60% decrease of VGluT1 IF boutons post-SCT was maintained in a stimulated hindlimb (p = 0.008, Mann-Whitney U-test; as compared to control values) and was not significantly different from a decrease in sham-stimulated hindlimb (p = 0.14, Wilcoxon test). A 34% decrease of density of VAChT IF boutons post-SCT (p = 0.008, Mann-Whitney U-test; as compared to the control values) was maintained in the stimulated hindlimb, and did not differ significantly from the decrease, which developed in sham-stimulated hindlimb (p = 0.27, Wilcoxon test; Fig 4). After stimulation, a detected tendency to higher volume of VAChT IF boutons (p = 0.068, Wilcoxon test; Fig 4) was confirmed in sized analysis, where an increase in one class of sized terminals (vol. between 15–19 μm^3^) occurred (Fig 5).

### Does stimulation of low-threshold proprioceptive afferents alter expression of glutamatergic AMPA and NMDA and cholinergic M2 receptors in LG α-motoneurons in spinal rats

#### Laser microdissection of LG and TA motoneurons

**Fig 6:**
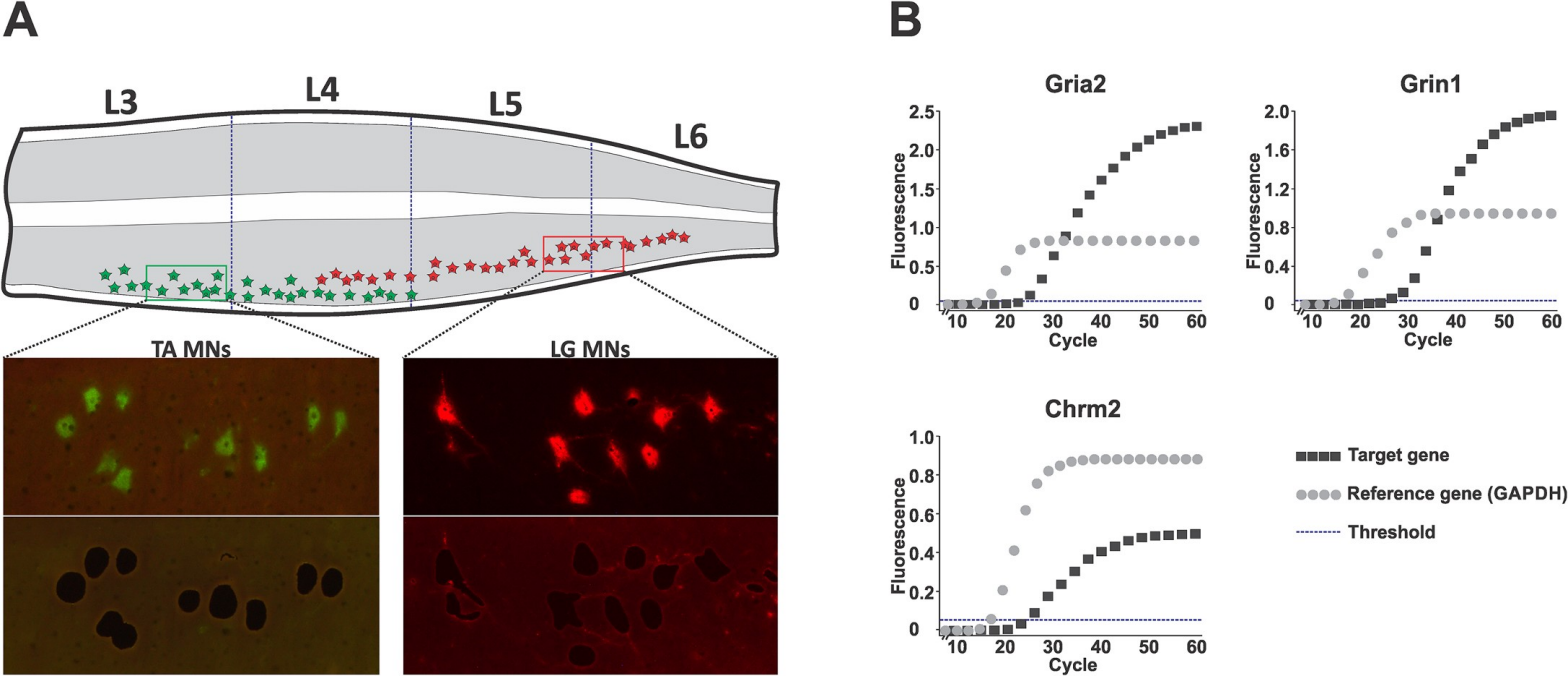
The expression of glutamatergic receptors (NMDA NR1 (*Grin1*) subunit, AMPA GluR2 (*Gria2*) subunit), M2 muscarinic cholinergic (*Chmr2*) receptor, and GAPDH genes in microdissected LG and TA motoneurons. **A**. Scheme of LG and TA distribution in L3-6 lumbar segment and representative photomicrographs showing LG and TA MNs retrogradely labeled by intramuscular injection of Alexa Fluor 555- (LG) and Alexa Fluor 488-conjugated cholera toxin subunit B (TA). Lower panel: confirmation of complete capture of traced LG and TA MNs by lack of fluorescence in the selected sections of spinal cord tissue. **B**. Real-time RT-PCR representative amplification curves for each transcript across control groups are shown. Relative quantification of receptor expression was performed using the 2^-ΔΔCt^ method data analyses. The cycle number at which the amplification curve crossed the threshold line determines threshold cycle (Ct). Expression for each sample and transcript was found by the difference (ΔCt) between the Ct for receptor transcript and GAPDH.

LG and TA motoneurons were localized in a rostro-caudal column within the grey matter of the lumbar spinal cord segments L3-L6, as described previously [31]. Visual inspection of the remaining spinal cord tissue after LMD confirmed complete capture of the traced LG and TA neurons (Fig 6). The numbers of LG and TA MN sections collected from the control (CN) and spinal (SCT) groups of animals were comparable. An average of 371 +/- 22 (LG-CN), 339 +/- 7 (TA-CN), 347+/- 37 (LG-SCT) and 333 +/- 13 (TA-SCT) 20 μm thick MN sections were captured per animal (mean +/- SEM). Because MN diameter ranges from 20–50 μm, that corresponds to 150–350 LG and TA MN collected per animal.

#### Glutamatergic and muscarinic receptor expression in LG and TA motoneurons

Expression of glutamatergic receptors [NMDA NR1 (*Grin1*) subunit, AMPA GluR2 (*Gria2* subunit], M2 muscarinic receptor (*Chrm2*) and reference *GAPDH* gene in microdissected MNs was quantified using duplex real-time RT-PCR. Representative amplification curves for all transcripts are shown in Fig 6B. Animals in both experimental groups showed detectable mRNA expression level.

**Fig 7:**
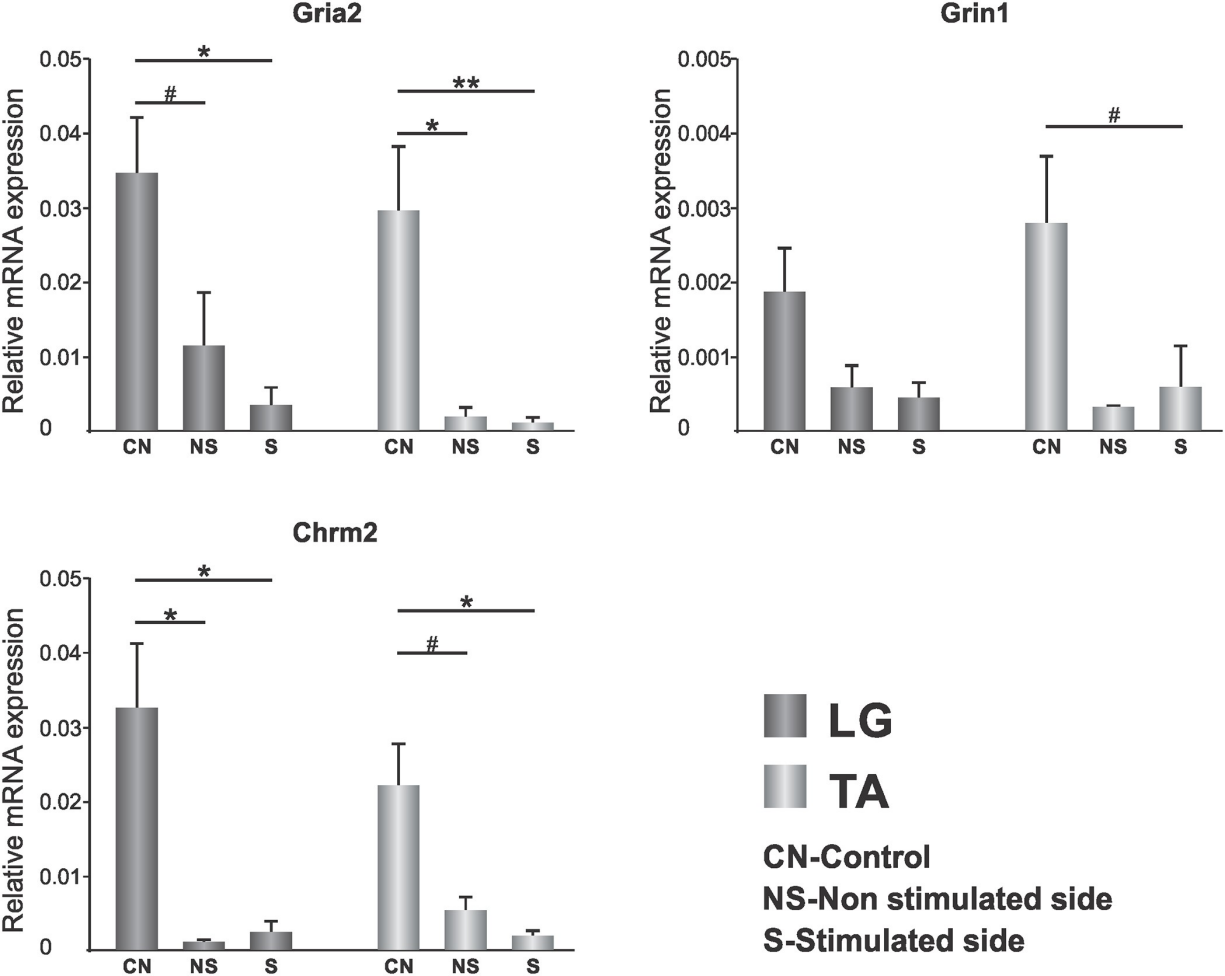
Changes in the expression level of AMPAR GluR2 (*Gria2*), NMDAR NR1 (*Grin1*), subunits and M2 muscarinic receptor (*Chrm2*) transcripts in LG and TA MNs. The effect of SCT and 7 days of low-threshold stimulation of proprioceptive fibers in the tibial nerve is shown. Data are means +/- SEM from 9 CN and 6 SCT-S rats. **p < 0.*01*, *p<0.*05*, *Mann-Whitney U test*.

In control samples mean Ct for the target genes was 24 (*Gria2*), 25 (*Chrm2*) and 29 (*Grin1*), and for the reference gene 18. It indicates that AMPA GluR2 (*Gria2*) subunit and M2 receptor (Chrm2) transcript levels were of the same order of magnitude, and were approximately 10 times higher from the level of expression of NMDA NR1 (*Grin1*) subunit (Fig 7). Both glutamatergic receptors and M2 receptor were expressed in LG and TA MNs at the comparable levels, indicating that transcriptional potential of genes coding for these excitatory receptors or their turnover is similar in ankle extensor and flexor MN pools in physiological conditions.

SCT in rats which did not receive electrodes implantation caused a profound decrease of M2 receptor (Chrm2) NMDA and NR1 (Grin1) subunit transcript levels (by 80–90%) both in LG and TA MNs, while AMPA GluR2 (Gria2) subunit transcript change was less (by 20%), but revealed to a similar extent in LG and TA MNs.

LG and TA MN expression of AMPA GluR2 subunit was significantly decreased after SCT in implanted rats (LG- threefold; p = 0.06, and TA—fifteenfold; p = 0.01). LG and TA MN expression of NMDA NR1 subunit tended to decrease (LG—threefold; TA—tenfold, p = 0.05); LG and TA MN expression of M2 receptor was also significantly decreased (LG-30 fold; p = 0.01, TA—fourfold; p = 0.08).

Seven days of enhancing direct Ia-signaling to LG MNs did not have significant influence on the expression level of any of the receptors under study neither in LG nor in TA MNs.

## Discussion

The aim of the presented experiment was to enhance synaptic Ia proprioceptive input to the MNs innervating the ankle extensor muscles, which were found to be particularly vulnerable to the complete spinal cord transection (SCT) several weeks thereafter [1, 9]. To counteract a reduction of movement-related proprioceptive input to MNs of unloaded ankle extensor muscles in spinal paraplegic animals, the continuous burst stimulation of the tibial nerve, which started two days after SCI, was continued for the next 7 days. The current was controlled by means of H-reflex and threshold M-responses recorded in the soleus muscle. This experimental strategy has been chosen to intervene into the processes involved in spontaneous reorganization of the spinal neuronal network, which starts shortly after spinalization [32].

Increased frequency of complex R2 and R3 responses to the burst stimulation indicate, that the H-reflex loop was activated and its effectiveness increased during consecutive days of this stimulation. The continuous bursts were delivered at 40Hz during four 20 min stimulation sessions daily, securing quite intensive activation of low-threshold proprioceptive fibers in the tibial nerve. This stimulation, effective in the intact rats, was primarily addressed to direct glutamatergic innervation of LG MNs by Ia terminals which were identified with VGLUT 1 IF antibody specifically labeling them [9]. Here we show that the same stimulation paradigm was not sufficient to prevent an impoverishment of glutamatergic and, to lesser extent, cholinergic innervation of LG MNs after SCT. However, a dichotomy in the effects of SCT on the density of VGLUT 1 IF boutons suggests, that the stimulation tended to increase the number of VGLUT1 IF boutons only in those animals which suffered of deep reduction of these inputs after SCT.

One of the important factors, which could limit the effectiveness of applied stimulation, was its delivery period, largely during the “spinal shock” or rather areflexia period. The duration of the “spinal shock” symptoms, which appear immediately after spinal cord injury, varies depending on the species and severity of the injury [33]. In rats subjected to SCT at low-thoracic segments the spinal shock lasts at least 2 days after surgery, as reasoned upon the heat shock proteins (HSPs 27) level, shown to profoundly increase in the lumbar spinal cord on the 2^nd^ post-surgery day and normalize on the 5^th^ day after SCT [34]. Both soleus muscle and spinal cord responses to exercise change 5 days after SCT suggesting that at that time point the animals recovered from the spinal shock [34]. In our experiment the complex (R2 and R3) responses to the burst stimulation gradually increased indicating that the proprioceptive input to MNs under study tended to increase in time after SCT. It is also possible that a suppression of the proprioceptive input to LG MNs might be related to the increased pre-synaptic inhibition of primary afferents after SCT as reported by Quevedo and co-authors [35]. The diminished receptiveness of MNs to proprioceptive stimuli might stem from a dysfunction and reduced expression of the glutamatergic and cholinergic receptors in that period, as shown in this study. For the purpose of the current study we applied the advanced method of LMD to strengthen the selectivity of readout from MNs population and examine receptor responses at the gene expression level in single MN type. While the revealed drops in the transcript level in LG MNs do not necessarily reflect similar changes in the production of receptor protein and the insertion of such into the membrane, they may still serve as an indicator of altered receptor production. We documented recently [36] and confirm here using that selective approach, that at the second week after SCT, the levels of muscarinic M2 receptor transcript are reduced in the L3–6 segments. Muscarinic receptor binding properties are also decreased in the L5–6 segments, where ankle extensor MNs are predominantly located; since these decreases were accompanied by M2 receptor protein redistribution between synaptic and extrasynaptic areas on MNs surface, the possibility remains that this type of compensatory responses develop also after stimulation and may influence MN excitability [36]. Moreover, because muscarinic M2 receptors are localized not only at the cholinergic but also at the glutamatergic (Glu) synapses [37], and control AMPA receptor responsiveness [38], their changed expression may contribute to altered glutamatergic signaling and excitatory properties of MNs. An individual analysis of the amplitude of complex responses gave us an indication of increased excitability of the H-reflex loop. Unfortunately, complementary analysis of Glu receptors at the protein level was not done because of unsatisfactory quality of antibodies designed to recognize single subunits of the Glu receptors under study.

An important factor in these considerations is the transient disappearance of voltage-activated sodium and calcium persistent inward current (PIC) in MNs, which was observed shortly after SCT [39]. The latter led to a pronounced reduction of the excitability of MNs, limiting their ability of amplifying the synaptic input to MNs to secure their sustained depolarization [40].

This early post-acute period, may not be indicative of how the system will respond at later time points. Some compensatory phenomena, which develop weeks after SCT, may form the basis to extend the application of that demanding approach, used in the current study [36]. Using higher-threshold stimulation addressed also to the group II muscle afferents in the tibial nerve should be considered. In the experiments speaking in favor of such possibility the current applied to the sciatic nerve initiated the contraction of the soleus muscles in the cat [41]. When that stimulation was delivered during first 8 days after SCT it not only prevented shortening of duration of afterhyperpolarization of soleus MNs but also prevented the loss of weight of this muscle.

Our quantitative analysis of glutamatergic Ia and cholinergic inputs to LG and TA MNs carried in this experiment provided evidence, which extends our understanding of the behavior of their preserved inputs and vulnerability of extensor and flexor MNs to SCT. Contrary to selective loss of inputs and dysfunction of extensor MNs developing at 6 weeks after transection [1, 9, 36], here we show an early and profound reduction of glutamatergic and cholinergic inputs to both extensor LG and flexor TA MNs. Moreover, we show that presynaptic changes are accompanied by reduced expression of AMPA *GluR2* and NMDA *NR1* receptor subunits and *M2* muscarinic receptor, similarly in LG and TA MNs. Importantly, our finding on *GluR2* loss of transcripts suggests a relative increase of GluR2-lacking, calcium-permeable AMPA receptors (CP-AMPARs) on MNs, that, if confirmed at the protein level, would be in line with reports on that SCI specifically increases postsynaptic localization of CP-AMPARs on them [42]. Functionally, AMPAR activity was reported to impact spinal cord motor training, suggesting potential for dynamic modulation of AMPAR and induction of synaptic plasticity in motor nuclei [43]. Peripheral stimulation below the injury was shown to engage CP-AMPAR-mediated Ca (2+) influx, activating intracellular modulators of synaptic plasticity and strengthening excitatory tone to promote adaptive spinal training. However, CP-AMPARs are hyper-responsive to peripheral input and are easily overdriven, what may result in synaptic saturation that overwhelms the capacity for adaptive spinal cord learning [22]. Modulation of these receptors might be an attractive therapeutic target.

Results collected in this study show, that at the early postlesion period, within a week post-SCT, both groups of MNs respond with loss of inputs and downregulation of their receptors, revealing similar vulnerability to the lesion and suggesting similar impairment of excitatory signaling to them at that postlesion period. Changes, which develop in time and differentiate their state at later period, have the underpinning in their differential functional conditions (unloading of ankle extensor but not flexor muscles) but may also result from differences in their intrinsic properties [31, 36]. Our recent study suggested that with time, extensor (Soleus) and flexor (TA) MNs develop a common (albeit revealed in extensor MNs to a lesser extent) compensatory mechanism in response to reduced strength of cholinergic signaling [36]. As already mentioned, this mechanism, operating at the protein level, is based on enrichment in synaptic vs extra-synaptic M2 receptors, and increased cytoplasmic M2 receptor content after SCT. Whether extensor MNs compared with flexor MNs are more responsive to activity-based interventions long-term post-transection than at early period, and respond differentially at the level of glutamatergic receptor proteins, requires further studies.

## Supporting information

S1 Table Frequency of appearance of R1 (M1) responses in single animals. The occurrence of M1 responses was counted during 3 s time intervals and is presented as a percentage of 118 bursts of stimuli. Counting started at 70, 600 and 1150 seconds of the first training session on days 1 and 7.
(DOCX)

S1 Fig The photos taken from oscilloscope recordings made in intact animals subjected to the train paradigm of stimulation (described in details in Materials & methods section and exemplified in Fig 2A). Light green lines indicate the most recent responses; darker green lines indicate superimposed responses to the number of preceding stimuli (we usually worked with averaging after 64 repetitions). A-C. Clear recruitment of H-reflex with small and variable (A) or none (B,C) M1.
(DOCX)
