# Supplementary material for: Early pre- and postsynaptic decrease in glutamatergic and cholinergic signaling after spinalization is not modified when stimulating proprioceptive input to the ankle extensor α-motoneurons: Anatomical and neurochemical study
Source: PLoS One. 2019 Sep 26;14(9):e0222849. doi: 10.1371/journal.pone.0222849 (PMC6763201; doi:10.1371/journal.pone.0222849)
Supplement: S1 Table — The occurrence of M1 responses was counted during 3 s time intervals and is presented as a percentage of 118 bursts of stimuli. Counting started at 70, 600 and 1150 seconds of the first training session on days 1 and 7. (DOCX) [file pone.0222849.s001.docx]

**PONE-D-19-06921R3**

**Grycz et al. Supplementary Table**

**S1 Table.** **Frequency of appearance of R1 (M1) responses in single animals.**

The occurrence of M1 responses was counted during 3 s time intervals and is presented as a percentage of 118 bursts of stimuli. Counting started at 70, 600 and 1150 seconds of the first training session on days 1 and 7.

| Rat  no. | day 1 session 01 | | | day 7 session 01 | | |
| --- | --- | --- | --- | --- | --- | --- |
|  | 70s | 600s | 1150s | 70s | 600s | 1150s |
| Ba1 | 22 | 12 | 8 | 8 | 8 | 3 |
| Ba2 | 1 | 2 | 3 | 3 | 10 | 0 |
| Ba3 | 2 | 0 | 0 | 7 | 10 | 7 |
| Ba5 | 16 | 4 | 19 | 78 | 15 | 11 |
| Ba6 | 1 | 2 | 1 | 3 | 7 | 8 |
| Ba8 | 5 | 14 | 5 | 24 | 27 | 33 |
