# Supplementary material for: Early pre- and postsynaptic decrease in glutamatergic and cholinergic signaling after spinalization is not modified when stimulating proprioceptive input to the ankle extensor α-motoneurons: Anatomical and neurochemical study
Source: PLoS One. 2019 Sep 26;14(9):e0222849. doi: 10.1371/journal.pone.0222849 (PMC6763201; doi:10.1371/journal.pone.0222849)
Supplement: S1 Fig — Light green lines indicate the most recent responses; darker green lines indicate superimposed responses to the number of preceding stimuli (we usually worked with averaging after 64 repetitions). A-C. Clear recruitment of H-reflex with small and variable (A) or none (B,C) M1. (DOCX) [file pone.0222849.s002.docx]

**PONE-D-19-06921R3**

**Grycz et al. Supplementary Figure**

**S1 Figure.** The photos taken from oscilloscope recordings made in intact animals subjected to the train paradigm of stimulation (described in details in Materials & Methods section and exemplified in Fig 2A). Light green lines indicate the most recent responses; darker green lines indicate superimposed responses to the number of preceding stimuli (we usually worked with averaging after 64 repetitions). A-C. Clear recruitment of H-reflex with small and variable (A) or none (B,C) M1.


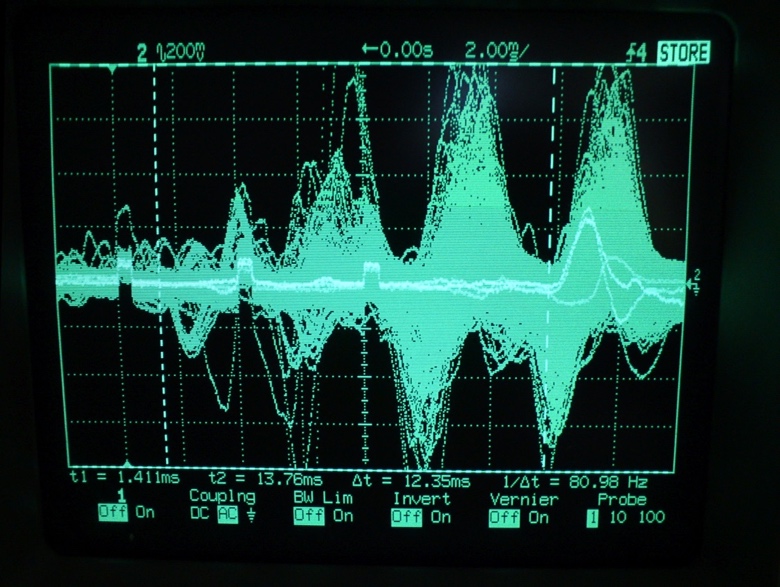

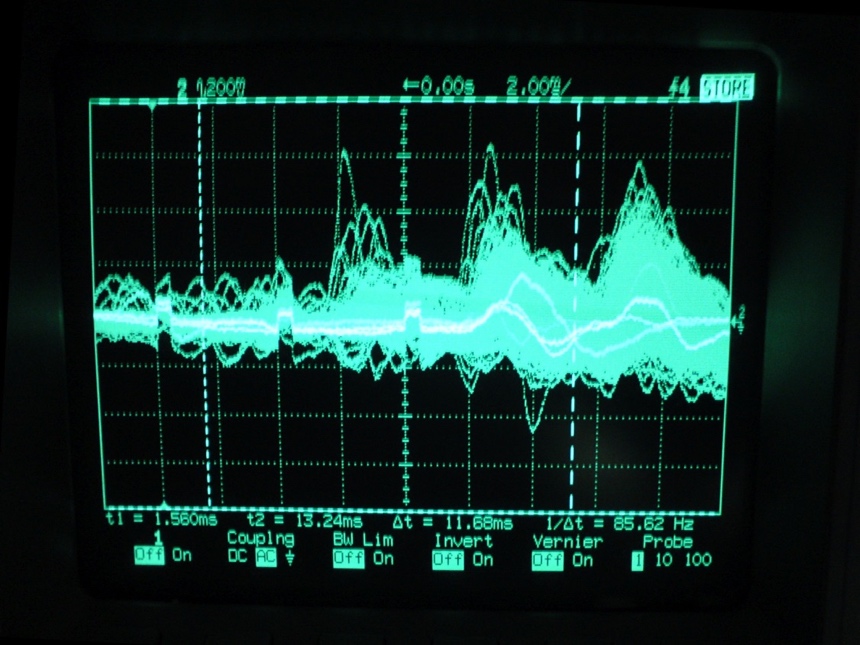


**B**

**A**


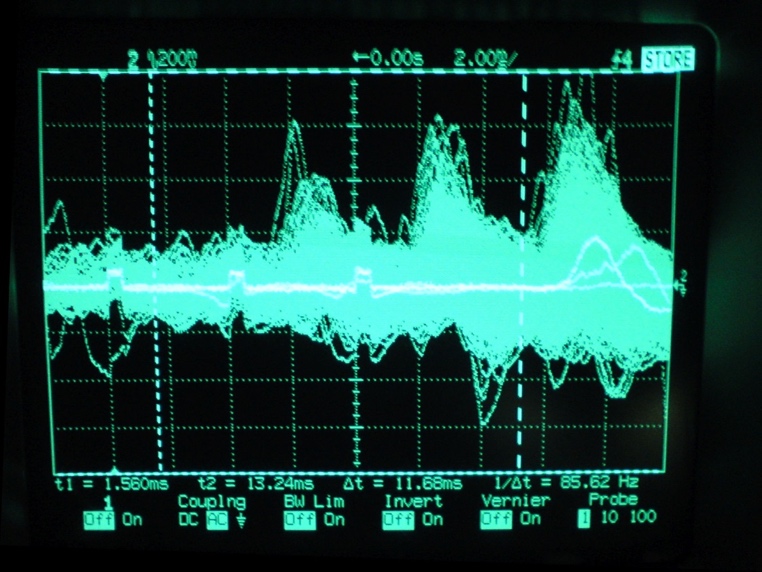


**C**
